# Metabolism, Physiological Role, and Clinical Implications of Sphingolipids in Gastrointestinal Tract

**DOI:** 10.1155/2013/908907

**Published:** 2013-09-05

**Authors:** Krzysztof Kurek, Bartłomiej Łukaszuk, Dominika M. Piotrowska, Patrycja Wiesiołek, Anna Małgorzata Chabowska, Małgorzata Żendzian-Piotrowska

**Affiliations:** ^1^Department of Physiology, Medical University of Bialystok, Mickiewicza Street 2C, 15-222 Białystok, Poland; ^2^Department of Public Health, Medical University of Bialystok, Szpitalna Street 37, 15-295 Białystok, Poland; ^3^Regional Blood Center, M. Skłodowskiej–Curie Street 23, 15-950 Białystok, Poland

## Abstract

Sphingolipids in digestive system are responsible for numerous important physiological and pathological processes. In the membrane of gut epithelial cells, sphingolipids provide structural integrity, regulate absorption of some nutrients, and act as receptors for many microbial antigens and their toxins. Moreover, bioactive sphingolipids such as ceramide or sphingosine-1-phosphate regulate cellular growth, differentiation, and programmed cell death—apoptosis. Although it is well established that sphingolipids have clinical implications in gastrointestinal tumorigenesis or inflammation, further studies are needed to fully explore the role of sphingolipids in neoplastic and inflammatory diseases in gastrointestinal tract. Pharmacological agents which regulate metabolism of sphingolipids can be potentially used in the management of colorectal cancer or inflammatory bowel diseases. The aim of this work is to critically the review physiological and pathological roles of sphingolipids in the gastrointestinal tract.

## 1. Introduction

**Figure 1:**
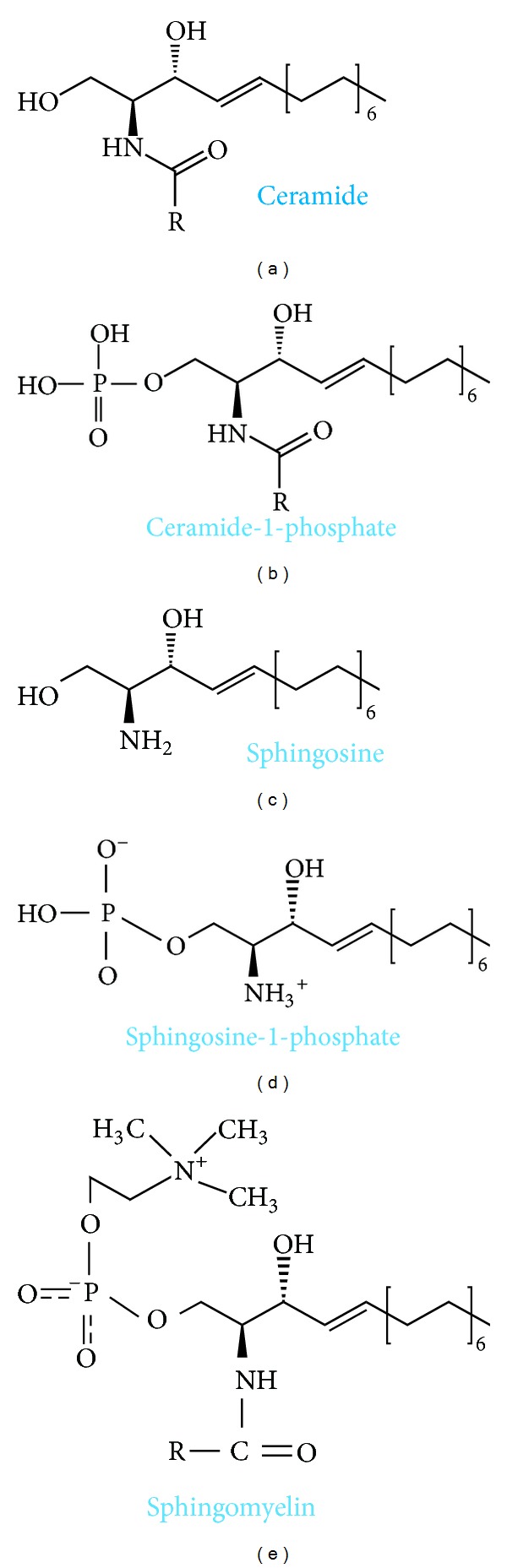
Biochemical structure of selected sphingolipids.

**Figure 2:**
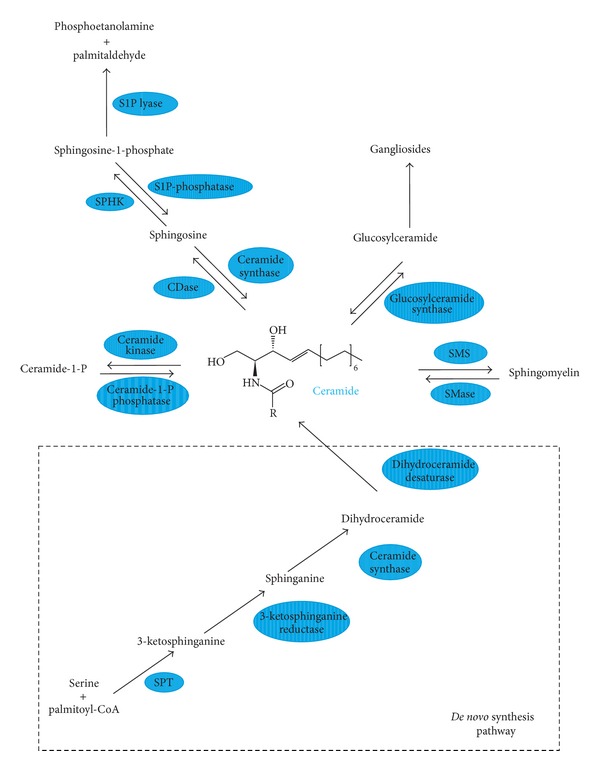
Schematic pathways of sphingolipids metabolism. CDase: ceramidase; S1P lyase: sphingosine-1-phosphate lyase; S1P phosphatase: sphingosine-1-phosphate phosphatase; SMase: sphingomyelinase; SMS: sphingomyelin synthase; SPHK: sphingosine kinase; SPT: serine palmitoyl transferase.

Sphingolipids (Figure 1) are a group of lipid organic molecules, composed of sphingoid base and free fatty acids residues. They were first described almost 130 years ago in 1884 [1], and nowadays this class encompasses sphingomyelin (SM), ceramide, and glycosphingolipids. Many of them serve as a structural component of cellular membrane. Moreover, sphingolipids play a significant role in the intracellular signal transduction. Sphingosine (SPH) makes up the backbone of all sphingolipids. The condensation of SPH and free fatty acid forms ceramide. Ceramide, in turn, can be combined with phosphocholine to form plasma membrane sphingomyelin as well as with neutral or acidic sugar residues to produce glycosphingolipids. Glycosphingolipids linked with sialic acid are called gangliosides (GM) (Figure 2). The major molecule in the pathway of sphingolipid signal transduction is ceramide, which regulates numerous cellular processes, including cellular proliferation, differentiation, and programmed cell death. Ceramide derivatives, ceramide-1-phosphate (C1P), sphingosine, and sphingosine-1-phosphate (S1P), have also bioactive properties. Herein, we discussed physiological role and clinical implications of sphingolipids in gastrointestinal tract.

## 2. Physiological Role of Sphingolipids in Gastrointestinal Tract 

### 2.1. Presence of Sphingolipids in Digestive System

Sphingolipids comprise just about 30–40% of all lipid fractions, present in digestive system, and were isolated from liver and pancreas parenchyma, as well as from mucosal cells of gastrointestinal tract. These lipids are expressed in small intestine mucosal cells, where the level of sphingolipids is over twofold higher than in colonic mucosa [2]. These differences are the result of excessive and rapid differentiation and exfoliation of mucosal cells in the upper gastrointestinal tract. Estimated sphingolipids daily requirement for gastrointestinal mucosal recovery is about 1.5 g [4]. In the intestinal villi, sphingolipids are located mainly in the apical membrane and in minor extent in the basolateral membrane [5]. Mucosa of small intestine is particularly rich in SM, ceramide, and glucosylceramide. The stomach mucosa, especially the secretory membrane where proton (K^+^/H^+^ ATPases) pumps are located [6], contains SM and gangliosides [7]; however, the role of mentioned sphingolipids in the stomach remains elusive. The protective role of gangliosides in the acidic environment has been postulated [8]. Ganglioside GM3 is the most abundant in the small intestine mucosa. Sphingolipids are delivered to the mucosal cell with diet or are synthesized via *de novo* pathway (Figure 2) [2]. 

### 2.2. Metabolism of Sphingolipids in Gastrointestinal Tract

As mentioned above, sphingolipids in gastrointestinal tract are synthesized mainly in *de novo* pathway, where first reaction, catalyzed by serine palmitoyl transferase (SPT), is condensation of amino acid serine with palmitoyl-CoA. This enzyme is commonly expressed in many tissues including liver, pancreas, and gastrointestinal tract mucosa. The product of described reaction, 3-ketosphinganine, is quickly reduced to sphinganine and further acylated to dihydroceramide. In the last step of *de novo* synthesis, ceramide is desaturated by dihydroceramidase desaturase (ceramide synthase), which introduces one double bond between C4 and C5 positions in sphingoid core [9]. As a result, dihydroceramide is converted to ceramide. Interestingly, five out of total six isoforms of ceramide synthases, except the third one, were found in the intestinal mucosal cells [10]. 

Another plausible pathway of ceramide synthesis is hydrolysis of plasma membrane sphingomyelin. This reaction is catalyzed by sphingomyelin phosphodiesterase (sphingomyelinase—SMase). So far, three isoforms (acidic, neutral, and alkaline) of sphingomyelinases were isolated from gastrointestinal mucosa. 

An alternative way of ceramide synthesis is described by Kitatani et al. [11], so called “salvage pathway,” which is based on its formation from free sphingosine [11]. Moreover, ceramide, located in the center of sphingomyelin signaling pathway, can be also phosphorylated to ceramide-1-phosphate or deacylated to sphingosine. Bioactive sphingolipid sphingosine-1-phosphate is a product of sphingosine phosphorylation reaction catalyzed by sphingosine kinase [12]. 

Although expression of all enzymes catalyzing sphingolipids metabolism in digestive system was described, the activities of individual enzymes vary in different organs. The SPT activity is highest in the liver, followed by stomach, small intestine mucosa, and pancreas [13]. In mucosal cells of small intestine, expressions of neutral SMase and alkaline SMase (enzymes catalyzing SM hydrolysis in optimal and alkaline pH, resp.) were identified [14]. Studies by Duan [15] proved that alkaline SMase is also present in human liver and pancreas, and it is released into the gut by bile salt or pancreatic juice [15]. Three isoforms of ceramidases (acidic, neutral and alkaline), responsible for transformation of ceramide into SPH, were identified in the intestinal mucosa. Among them is alkaline ceramidase, which catalyzes reaction in alkaline environment in the presence of bile salts of taurocholic and taurochenodeoxycholic acids and is characterized by the highest catalytic activity [16, 17]. Moreover, neutral ceramidase occurs also in human liver [18]. Both sphingomyelinases and ceramidases belong to the group of ectoenzymes, situated on the outer surface of a cell's membrane so that their active sites are available to the exterior environment of the cell. Because of that, SMases and ceramidases are able to catalyze hydrolysis of SM or ceramide inside mucosal cells as well as in the lumen of the gut [19]. Besides that, part of ceramide in the gut is hydrolysed via activation of (bile salt stimulated lipase) present in pancreatic juice BSSL [20]. However, in BSSL knock-out mice (−/−), digestion of ceramide was not decreased [21], so it can be concluded that more important enzyme catalyzing ceramide hydrolysis is neutral ceramidase. Expression of discussed enzymes is affected by some drugs. For example, activity of alkaline sphingomyelinase is increased by ursodeoxycholic acid, anti-inflammatory substances (e.g., 5-ASA), and psyllium (dietary fiber supplement used in the treatment of constipations) [16]. These factors simultaneously decreased ceramidases activities, thus leading to ceramide accumulation in the gut [22]. Nonetheless, activity of alkaline sphingomyelinase is decreased by a high fat diet feeding [15]. Expression of sphingosine kinase in gastrointestinal tract has also been proven. In small intestinal and colonic mucosal cells, sphingosine kinase type 1, and in a lesser extent also type 2, is present [23]. Despite that, the level of S1P in the gut is relatively low because this molecule is quickly degraded, by presence in mucosal cells S1P lyase, to phosphoetanolamine and palmitaldehyde [24]. 

### 2.3. Sphingolipids in Diet

Daily dietary intake of all sphingolipids in adult human is estimated about 300–400 mg [25]. Fruit and vegetable products provide only about 50 mg of sphingolipids per day. Especially rich in sphingolipids are dairy products, particularly eggs and milk. Human milk is the only source of sphingolipids for neonates and it consists of SM, lactosylceramide, glucosylceramide, and gangliosides (GM1, GM3, and GD3). An infant who drinks ca. 700 mL of milk daily ingests about 119 *μ*mol of SM [26]. Among mentioned lipids, gangliosides are the most significant since they contribute to proper central nervous system growth and inactivation of some microorganisms in the gut during the infancy [27]. Nursling consumes averagely about 50–150 mg of SM daily. It is important to emphasize that commercial bovine milk, as well as soy protein-based infant formulas, has very low levels of SM and gangliosides, almost twice lower, compared to human milk [28]. Accordingly, feeding infants with the commercial available bovine milk may result in the abnormal sphingolipids content in gut, leading to long-term consequences, such as immunodeficiencies or abnormal development of central nervous system [29–31]. Some sphingolipids, except SM and gangliosides, are present in fruit and plants (cucumbers, grapes, broccoli, black bean, and wheat). Interestingly, rates of digestion and absorption of vegetal sphingolipids in the gut are lower compared with animal-origin ones [32]. Another major source of sphingolipids, mainly SM, animal-origin tissues like poultry (chicken, turkey), beef, pork, and fish (salmon, catfish) [33]. 

As mentioned above SM is digested and absorbed mainly in the small intestine. Animal studies proved that consumed SM is digested only partially and it is a slow process [34]. On the other hand, in human more than 80% of SM can be digested, and the rest is excreted with feces [35]. SM is resistant to digestion by pancreatic enzymes [2]. On the other hand, another sphingolipids sphingosine and dihydrosphingosine are quickly absorbed in the small intestine and further metabolized to free fatty acids, mainly palmitate, and in the lesser extent to ceramide. In summary, sphingolipid profile in the gut depends on dietary components and sphingolipid intake may influence their amount in the intestinal mucosa. Interestingly, intestinal microflora has no significant effect on the sphingolipid content in the gut [36]. 

### 2.4. Role of Sphingolipids in Binding and Inactivation of Toxins and Bacteria

Sphingolipids are responsible for proper gastrointestinal tract function. Gangliosides which are profusely present on the surface of the apical membrane of enterocytes protect intestinal mucosa from injury by bile salts [5]. They also function as binding sites for bacteria and their toxins to prevent translocation of pathogens from the gut to the internal environment. Bacteria, viruses, and toxins are inactivated after binding with glycosphingolipids, and by that exogenous sphingolipids (provided in diet) protect passage of the microorganisms through the intestinal mucosa. For example, bacterial toxins of *Shigella* and *Escherichia* or rotaviruses are bound and inactivated [37]. GMs, negatively charged glycosphingolipids, are able to bind some pathogens and their toxins. It has been proven that GM1 binds and inactivates toxins of *Vibrio cholerae* and heat-labile toxin of *Escherichia coli* [38, 39]. Furthermore, GM3 binds rotaviruses and enterotoxigenic *Escherichia coli* [40, 41]. The majority of microbial toxins induce inflammation in the gut, manifested primarily by nausea, vomiting, abdominal pain, and diarrhea. Accordingly, proper gangliosides supplementation, for example, by consumption of milk, eggs, and other dairy products may protect from infections through binding and inactivation of bacterial toxins [42, 43]. Idota et al. [44] demonstrated that in cases of breast milk fed infants GMs inhibit toxins of *E. coli* and *Vibrio cholerae*. Moreover, present in human milk gangliosides can stimulate growth of probiotic bacteria strains, such as *Bifidobacterium* [44]. Further studies proved that changes in intestinal microflora, manifesting in decreasing in *E. coli* and increasing in Bifidobacteria level, are promoted by ganglioside component—sialic acid [31]. Besides that, Suh et al. [45] showed in mice, that addition of GMs to the diet significantly reduced infection rate of protozoan *Giardia muris* which belongs to the same taxon as human intestinal pathogen *Giardia intestinalis* [45]. It has been found, in vitro, that sphingosine, but not ceramide, has potent antibacterial effect against intestinal pathogenic strains of *E. coli* O157:H7, *Salmonella enteritidis*, *Campylobacter jejuni,* and *Listeria monocytogenes* [46]. Therefore, it can be concluded that dietary sphingolipids, particularly milk and egg gangliosides, may protect gut against infections through binding and inactivation of microbes and their toxins. On the other hand, Lafont et al. [37] showed that toxic effect of *Shigella* toxin was significantly decreased in sphingolipid-deficient cell lines [37].

### 2.5. Role of Sphingolipids in Signal Transduction

Sphingolipids in gastrointestinal tract are engaged in signal transduction and regulate inflammation and mucosal cells proliferation, differentiation, or the process of programmed cell death (apoptosis). Ceramide, C1P, sphingosine, and S1P are the most important signaling molecules [47–49]. Ceramide and SPH are metabolites with antiproliferative and proapoptotic properties, which induce dephosphorylation and inhibition of proliferation and apoptosis protein kinases such as Akt, PKC, MAPK and PKC [12]. Interestingly, phosphorylation of ceramide and sphingosine to C1P and S1P changes diametrically the properties of these molecules. Phosphorylated derivatives of ceramide and SPH are characterized by remarkably proliferative and antiapoptotic properties. It is a result of modification of phospholipase A2, activation of protein kinases Akt and MAPK, and increased expression of cyclooxygenase 2 (COX2) by C1P and S1P leading to mucosal cells proliferation and inhibition of their apoptosis [50]. Sphingolipid disorders may result in abnormal mucosal cells proliferation, differentiation, and apoptosis in the gut, and, as a result, inflammatory and neoplasmatic digestive diseases are described later in detail.

### 2.6. Role of Sphingolipids in the Regulation of Intestinal Absorption Process

Present in brush border sphingolipids are able to regulate absorption of nutrient via activation of specific receptors. For example, sphingolipids in intestinal mucosal cells inhibit cholesterol absorption. Cholesterol absorption rate is decreased by the presence of dietary sphingomyelin in rats [51]. Interestingly, other studies revealed that milk SM is more effective in reducing cholesterol absorption than SM obtained from eggs [52]. This aforementioned inhibitory effect is a result of direct interaction between SM and cholesterol leading to a decreased cholesterol thermodynamic activity [53]. Moreover, Feng et al. [54] showed that cholesterol absorption is also inhibited by ceramide formed from SM through the activation of alkaline sphingomyelinase [54]. Those authors proved that ceramide, as an inhibitor of cholesterol absorption, is more effective than SM [54]. Moreover, cholesterol uptake by intestinal cells is suppressed by sphingosine, but it is less effective than SM and ceramide [55]. The above described findings allow to conclude that dietary sphingolipid supplementation leads to decreasing cholesterol absorption and could limit cholesterol-related diseases. 

## 3. Role of Sphingolipids in Selected Gastrointestinal Tract Diseases

### 3.1. Sphingolipids and Colorectal Tumorigenesis

In view of regulation of cellular proliferation, differentiation, and apoptosis by some sphingolipid metabolites, it is postulated, that they can have an important impact on tumorigenesis. It is well established that synthesized *de novo* or through SM hydrolysis ceramide and its derivative sphingosine have antiproliferative and proapoptotic properties. So it can be postulated that ceramide and SPH inhibit progression and growth of neoplasmatic cells. Decreased levels of these compounds were observed in lung, breast, ovary, liver, and neck cancers [56]. Moreover, it seems that in cancer cells increased ceramide glycosylation to glucosylceramide leads to decreasing ceramide level. Interestingly, Liu et al. [57] proved that ceramide glycosylation potentiates cellular multidrug resistance, including cytostatics in cancer tissue [57]. On the other hand, phosphorylated ceramide and SPH derivatives, C1P and S1P, have antiapoptotic properties; they may enhance cellular proliferation and increase angiogenesis. Increased levels of S1P and C1P were demonstrated to occur in many types of cancer in contrast to ceramide and SPH contents [58]. 

**Figure 3:**
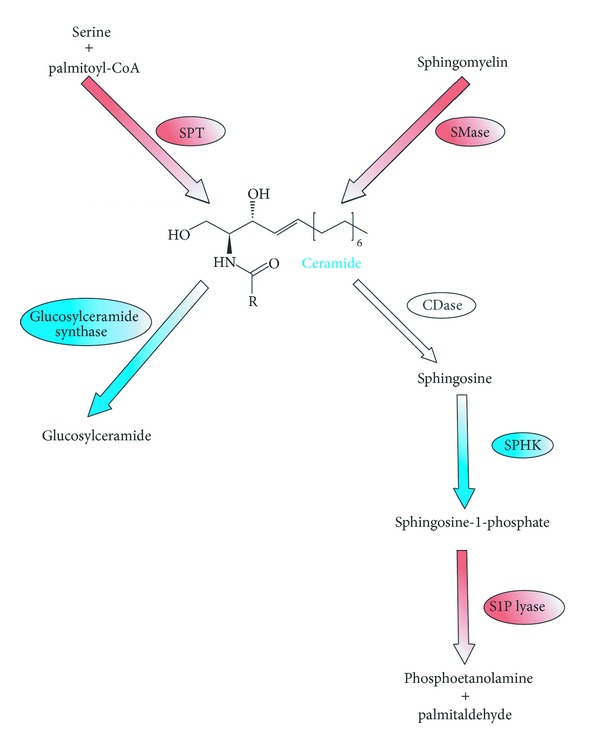
Changes (increase—blue color, decrease—red color) in activities of enzymes engaged in sphingolipids metabolism in colorectal cancer (adapted from [2, 3]). CDase: ceramidase; SMase: sphingomyelinase; S1P lyase: sphingosine-1-phosphate lyase; SPHK: sphingosine kinase; SPT: serine palmitoyl transferase.

The prospective role of sphingolipids in colon cancer development in rats treated with chemical carcinogen 1,2-dimethylhydrazine was first proposed by Dudeja et al. [59]. Those authors showed that SM level in colon cancer tissue was significantly increased [59]. Further studies by Dillehay et al. [60] revealed that dietary SM (both natural from bovine milk and synthetic forms) supplementation ensured relatively constant level of ceramide in colonic mucosal cells and prevented formation of aberrant crypt foci by 70% [60]. Another study showed that SM and ceramide levels in human colon cancer tissue are decreased compared to healthy patients [61]. Presented changes in sphingolipid levels are secondary to alterations in activities of enzymes regulating SM and ceramide metabolism (Figure 3). For example, alkaline SMase activity is decreased in human chronic colitis [62], colorectal cancer [63], and familial adenomatous polyposis [64] by 25%, 75%, and more than 90%, respectively. Furthermore, alkaline SMase was identified in the feces of patients with colorectal cancer, and its activity was significantly decreased compared to healthy ones [65]. Moreover, colon cancer tissue expresses abnormal SMase isoforms, which are totally inactive [66]. Reduction of SMase activity leads to decreased level of ceramide in patients with colorectal cancer. Interestingly, alkaline SMase can hydrolyze and inactivate platelet activating factor (PAF). Increased level of PAF was shown in inflammatory bowel diseases (IBDs) and neoplastic colon diseases, so it can be concluded that catalyzed by SMase PAF hydrolysis is favorable in these cases [36]. Besides that, alkaline SMase is able to hydrolyze lysophosphatidylcholine, which can promote the metastasis of colon cancer [67]. 

Sphingosine-1-phosphate is another sphingolipid which has an impact on colorectal carcinogenesis. As mentioned above, S1P has proliferative and antiapoptotic properties; therefore, it promotes neoplastic angiogenesis through the activation of platelet derived growth factor (PDGF) and vascular endothelial growth factor (VEGF) [58]. S1P could be considered as a cancerogenic prognostic factor since high level of this compound correlates with poor prognosis and survival rate in patients with glioblastoma multiforme [68]. It is possible that the same correlation exists in case of colorectal cancers. Increased level of S1P was observed in both human colon cancer tissues and in animals treated with azoxymethane (known from its carcinogenic properties). This is probably a result of upregulation of sphingosine kinase activity [69]. It has been documented that S1P acts by G protein-coupled receptors, localized on the plasma membrane. Furthermore, Müller et al. [70] showed the existence of intense upregulation of those receptors in human colon, breast, melanoma, and lung tumor cells [70]. Additionally, by using specific anti-S1P antibodies, inhibition of growth, invasion, and angiogenesis in multiple tumor lineages, including colorectal cancers, could be obtained [71]. Moreover, S1P expression is determined by activities of enzymes, which regulate its metabolism. In colorectal cancer cells, enzymes responsible for S1P degradation (S1P lyase and S1P phosphatase) are downregulated, so the catabolism of S1P is limited which results in S1P over accumulation [72]. In contrary, Oskouian et al. [73] showed that S1P lyase overexpression potentiates apoptosis via p53- and p38-dependent pathways in colon cancer [73]. Another important enzyme, engaged in sphingolipids metabolism in colorectal cancer, is sphingosine kinase, which catalyses phosphorylation of sphingosine to S1P. In the Min mouse (model of familial adenomatous polyposis), Kohno et al. [74] revealed that knocking out sphingosine kinase leads to decreased intracellular S1P level followed by significant reduction in adenomas size and inhibition of cell proliferation [74]. 

### 3.2. Sphingolipids and Intestinal Inflammation

As mentioned above, sphingolipids present in intestinal mucosa create nonspecific barrier and in that way protect enterocytes against digestive enzymes, bile salts, or acidic gastric juice. Dysfunction of these mechanism can result in the development and progression of inflammatory diseases. In porcine model, inhibition of ceramide *de novo* synthesis with mycotoxin fumonisin B1 alters the proliferation and barrier function of intestinal epithelial cells, which in turn leads to induction of inflammation [75]. In another study, Bock et al. [76] proved that exogenous sphingomyelinase causes deterioration of intestinal barrier function and increases inflammation due to reduction of SM in mucosal cells [76]. Furthermore, Furuya et al. [77] observed alleviated inflammatory bowel disease in experimental mice model after oral SM supplementation [77]. Another group of sphingolipids, gangliosides, are also characterized by their anti-inflammatory properties. For example, galactosylceramide inhibits ileitis, induced by *Toxoplasma gondii* infection, by overexpressing TNF-*γ* [78]. In contrary, S1P has strong proinflammatory properties; it activates neutrophils and macrophages and further induces mast cells degranulation. S1P also stimulates cyclooxygenase 2 (COX2), thus leading to production of inflammatory mediators [79]. Interestingly, orally administered sphingosine kinase inhibitors (ABC294640 and ABC747080) also cause S1P level reduction and significant improvement of DSS mice (model of ulcerative colitis) condition [80].

Another sphingolipid which can be engaged in pathogenesis of inflammatory and neoplastic bowel diseases is ceramide-1-phosphate. C1P promotes cellular proliferation and differentiation [81]. It also induces inflammation, acting as a positive allosteric activator of phospholipase A2 [82]. Moreover, C1P activates COX2 resulting in increased levels of prostaglandins, particularly PGE2, and plays an important role in the pathogenesis of inflammatory bowel diseases [83].

### 3.3. Role of Sphingolipids in Gastric Diseases and *H. pylori* Infection

Physiologically human gastric mucosa is characterized by relatively high level of gangliosides, higher even than in the intestinal mucosa [84]. This level is additionally increased in cases of stomach neoplasm. However, potential role of sphingolipids in gastric tumorigenesis is poorly investigated. It was evidenced that in gastric adenocarcinoma the level of GM2 is significantly elevated compared to normal gastric mucosa [85]. Another well-documented risk factor involved in gastritis, ulceration, and gastric carcinoma development is *Helicobacter pylori* infection. Some sphingolipids may serve as binding sites for *H. pylori* and their toxins. For example, lactosylceramide acts as adhesion receptor for *H. pylori* [86] and plasma membrane SM functions as receptor for *H. pylori* vacuolating toxin (VacA) [87]. Thus, hydrolysis of gastric SM by SMase decreased vacuolation induced by VacA [87]. Moreover, dietary SM supplementation, using bovine milk, inhibits adhesion of *H. pylori* to the gastric mucosa and reduces vacuolation [88]. On the other hand, Wada et al. [89] found that gangliosides are able to bind and neutralize *H. pylori* VacA toxin. In the discussed studies, oral administration of gangliosides resulted in regression of *H. pylori* infection [89]. Interestingly, neutral and acidic SMases were also indentified in *H. pylori* cells [90], but the potential significance of this phenomenon remains unexplained, although it may be related to gastric ulcers formation.

### 3.4. Role of Sphingolipids in Liver Cancer Pathogenesis

The role of sphingolipids in liver cancer pathogenesis is complex. In liver cancer cells, similarly as in a case of colon cancer, decreased ceramide level was observed. Reduction of ceramide level was a result of reduced activity of alkaline sphingomyelinase. Decreased activities of three types of SMases were found also in hepatic tissue samples obtained from patients with primary sclerosing cholangitis (PSC) which is precancerous condition and predisposes to cirrhosis and subsequent liver cancer development. Moreover, in liver cancer cells, defected isoforms of SMases, totally inactive, were identified [91]. It was also established that inhibition of ceramide *de novo* synthesis by fumonisin B1 induces liver cancer in rats. In studies of Gelderblom et al. [92], after 26 months of fumonisin B1 administration all rats developed cirrhosis and 66% of them developed hepatocellular carcinoma [92]. Fumonisin B1 is a mycotoxin, synthesized by *Fusarium* fungi, occurring in contaminated corn, sorghum, and grain, and it is potent and selective inhibitor of ceramide synthase [9]. Three-year studies of corn harvested in China revealed that fumonisin B1 is a risk factor for primary liver cancer and probably for esophageal cancer in humans [93, 94]. Higher incidence of liver cancer was presented in mice with decreased intrahepatocytes ceramide level. Most probably, it is a result of antiproliferative and proapoptotic properties of ceramide. Interestingly, ceramide derivatives galactosylceramide, alpha-glucosylceramide, and beta-glucosylceramide inhibit tumor metastasis in liver through the activation of neutral killer cells (NK), dendritic cells, and release of cytokines such as interleukin IL12 [95, 96]. On the other hand, lactosylceramide predisposes to multidrug, including cytostatics, resistance. However, ganglioside GD3 sensitizes human malignant hepatoma (hepatocellular carcinoma) cells to anticancer chemotherapy by inhibiting the activation of nuclear factor kappa-light-chain-enhancer of activated B cells (NF-*κ*B). Besides that, GD3 induces hepatoma cells apoptosis [97].

## 4. Summary and Future Perspective

It can be concluded that sphingolipids are important components of gastrointestinal tract. They exert numerous physiological functions and serve as receptors for microorganisms, and their toxins regulate intestinal absorption and participate in signal transduction. Besides that, sphingolipids have considerable clinical implications in numerous diseases, including gastrointestinal tumorigenesis and inflammation. Pharmacological agents aiming to regulate sphingolipid metabolism could be potentially used in the treatment of colorectal cancer or inflammatory bowel diseases in the future.
